# Investigations of the Laser Ablation Mechanism of PMMA Microchannels Using Single-Pass and Multi-Pass Laser Scans

**DOI:** 10.3390/polym16162361

**Published:** 2024-08-21

**Authors:** Xiao Li, Rujun Tang, Ding Li, Fengping Li, Leiqing Chen, Dehua Zhu, Guang Feng, Kunpeng Zhang, Bing Han

**Affiliations:** 1 Zhejiang Provincial Key Laboratory of Laser Processing Robotics, College of Mechanical and Electrical Engineering, Wenzhou University, Wenzhou 325035, China; 22461440079@stu.wzu.edu.cn (R.T.); 23461146041@stu.wzu.edu.cn (D.L.); zkp@wzu.edu.cn (K.Z.); 2 China International Science & Technology Cooperation Base for Laser Processing Robotics, Wenzhou University, Wenzhou 325035, China; 3 Institute of Laser and Optoelectronic Intelligent Manufacturing, Wenzhou University, Wenzhou 325035, China; 4 Oujiang Laboratory, Zhejiang Laboratory for Regenerative Medicine, Vision and Brain Health, Wenzhou 325035, China; lfp@wzu.edu.cn (F.L.); fengguang_516@126.com (G.F.); 5 Wenzhou Key Laboratory of Ultrafast Laser Precision Manufacturing Technology, Wenzhou 325035, China; 6 College of Mechanical and Electrical Engineering, Wenzhou University, Wenzhou 325035, China; zhu_556@163.com; 7 School of Electronic and Optical Engineering, Nanjing University of Science and Technology, Nanjing 210094, China; hanbing@njust.edu.cn

**Keywords:** microchannel, PMMA, laser machining, incubation effect, simulation model

## Abstract

CO_2_ laser machining is a cost effective and time saving solution for fabricating microchannels on polymethylmethacrylate (PMMA). Due to the lack of research on the incubation effect and ablation behavior of PMMA under high-power laser irradiation, predictions of the microchannel profile are limited. In this study, the ablation process and mechanism of a continuous CO_2_ laser machining process on microchannel production in PMMA in single-pass and multi-pass laser scan modes are investigated. It is found that a higher laser energy density of a single pass causes a lower ablation threshold. The ablated surface can be divided into three regions: the ablation zone, the incubation zone, and the virgin zone. The PMMA ablation process is mainly attributed to the thermal decomposition reactions and the splashing of molten polymer. The depth, width, aspect ratio, volume ablation rate, and mass ablation rate of the channel increase as the laser scanning speed decreases and the number of laser scans increases. The differences in ablation results obtained under the same total laser energy density using different scan modes are attributed to the incubation effect, which is caused by the thermal deposition of laser energy in the polymer. Finally, an optimized simulation model that is used to solve the problem of a channel width greater than spot diameter is proposed. The error percentage between the experimental and simulation results varies from 0.44% to 5.9%.

## 1. Introduction

Microfluidic devices have gained important applications in many areas, such as chemical synthesis [1], biology [2], and optofluidic technology [3], because of their advantages, like high sensitivity, rapid analysis, low sample and reagent consumption, and measurement automation, over conventional methods. Polymethylmethacrylate (PMMA) belongs to the class of thermoplastic polymeric materials. The inherent properties of PMMA, such as optical transparency, thermal stability, chemically inertness, biocompatibility, and low cost, have made it one of the most promising polymer materials for microfluidic devices [4].

Up to now, the microfluidic devices manufactured with PMMA generally are used in fuel cells [5], DNA analysis [6], blood detection [7], capillary electrophoresis [8], micro-reactors [9], and microchannel heat sinks [10]. These applications require 10–500 µm wide microchannels for the transfer, manipulation, and mixing of fluids. To fabricate the microchannels on PMMA, many manufacturing methods have been explored. Conventional methods such as micro-milling [11], hot embossing [12], and injection molding [13] are time-consuming and require many steps to obtain final microfluidics devices. Photolithography [14] and plasma etching [15] produce high-quality microchannels but involve several steps and special facilities, such as a clean room and sophisticated equipment. Moreover, the by-products of these processes are toxic in nature. In comparison to these techniques, the direct laser ablation provides a promising method for fabricating microchannels on polymers. The laser ablation method has various advantages like flexibility, ease in automation, simple steps in machining, less processing time, and lower cost in the fabrication of complicated geometric microchannels [16].

In laser ablation machining, bulk material is removed from the irradiated material surface by melting and vaporization. At present, CO_2_ and excimer laser have successfully processed the transparent polymer material because most of the polymers exhibit significant absorptivity at the mid-infrared spectrum and low UV wavelength [17,18,19]. In addition, femtosecond lasers also have been used to fabricate high-quality microchannels with precise sizes [20,21]. However, the femtosecond laser system requires expensive and highly sophisticated instruments and is associated with a significant cost of fabrication. Compared with UV laser and femtosecond laser systems, CO_2_ laser machining is a cost effective and time saving solution for fabricating microchannels on PMMA. CO_2_ laser systems provide a higher power output resulting in less processing time and higher ablation efficiency. Meanwhile, PMMA has a low heat capacity and a low heat conduction comparing other polymers, such as polycarbonate (PC) and polypropylene (PP) [22,23,24,25,26]. Therefore, most of the absorbed energy immediately causes the removal of local material without creating thermal cracks. Consequently, CO_2_ laser ablation results in clean and clog-free ablation on PMMA with a smaller heat affected zone (HAZ) and better surface finish [27].

CO_2_ laser fabricating microchannels on PMMA has been studied by various authors over the years. Chen et al. [28] investigated the influence of laser power, laser scanning speed, and scanning number on surface roughness of PMMA microchannel fabricated by a continuous CO_2_ laser. It was found that the scanning number had the greatest impact on the surface roughness. Bilican et al. [29] utilized a CO_2_ laser having a pulse duration of microseconds to fabricate microchannels on PMMA and PS substrates. It was observed that the size and profile of the microchannel could be changed based on laser power, scanning speed, pulse frequency, and defocusing distance. In order to investigate the influence of CO_2_ laser parameters on the profile of PMMA microchannels, Yuan et al. [30] derived a mathematical model based on the energy balance under a continuous Gaussian laser irradiation. Prakash et al. [31] studied the fabrication of PMMA microchannels using multi-pass CO_2_ laser processing based on experiments and numerical simulation. It was found that compared to a single-pass process, a multi-pass laser process produced a smaller channel width and a smaller HAZ. A model based on Yuan’s model was proposed, which was able to predict accurately the profile of the microchannel that undergone multiple passes. To investigate pulsed laser machining microchannels, Pazokian [32] used a simulation model of a ns laser ablating polymer that mainly considered the influence of laser spot overlap on channel depth. Meanwhile, other mathematical models [33,34,35] were utilized to analyze the ablation process of CO_2_ laser machining PMMA. These models considered that laser power was balanced by the increasing temperature of an infinitesimal volume of the polymer and the heat losses from decomposition and vaporization. Based on these considerations, the removal depth appeared to be proportional to the incident laser power and the pulse frequency and inversely proportional to the scanning speed. In addition, Anjum et al. [36] proposed a soft computing technique to build the prediction models of the channel depth, width, and surface roughness with multi-pass laser machine. By comparing various approaches, such as random forest, gradient boost, ridge regression, linear regression, support vector regression, and gaussian process regression, it was noticed that the physics-informed machine learning GPR technique could be used effectively in the depth, kerf width, and surface roughness estimations.

Up to now, most mathematical and simulation models mainly focus on predicting the variation in the channel depth with laser parameters, while considering that the channel width is approximately equal to the laser spot diameter. However, when using a high-power laser, the channel width is often greater than the laser spot diameter [34,35]. When the laser interacts with polymers, there is the incubation effect [37,38,39], which causes a decrease in the ablation threshold and an increase in the channel depth and width by increasing the pulse overlap and scanning number. Due to the lack of research on the incubation effect and ablation behavior of PMMA under high-power laser irradiation, the prediction of the microchannel profile is limited.

This paper investigates the ablation threshold of CO_2_ laser machining microchannels on PMMA using single-pass and multi-pass laser scan modes and analyzes the thermal decomposition behavior and incubation effect. Meanwhile, the influences of laser parameters on channel width, depth, volume ablation rate, and mass ablation rate are studied. Finally, a mathematical model is proposed to predict the profile of the microchannel based on the experimental results.

## 2. Experimental Set-Up and Procedure

**Figure 1:**
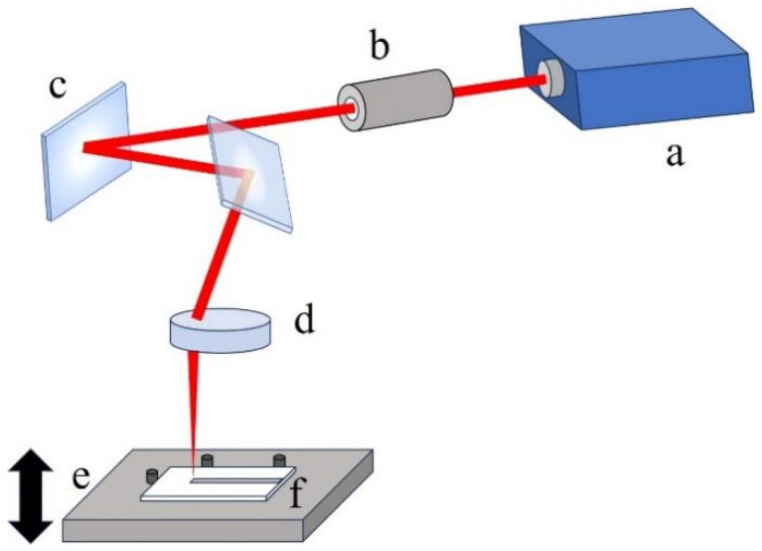
Schematic diagram of the experimental set-up: (**a**) CO_2_ laser source; (**b**) beam expander; (**c**) galvanometer systems; (**d**) telecentric f-theta lens; (**e**) adjustable platform; (**f**) PMMA sample.

A continuous CO_2_ laser system working at a constant output power of 7.8 W with a Gaussian profile was used to perform the experiments. The experimental set-up is schematically represented in Figure 1. The laser beam is concentrated into a minimum spot radius of 70 µm using a telecentric f-theta lens. A set of galvanometer systems is used to deflect the laser beam on the scanning surface. These movements are controlled using 2D CAD software, which allows the machining path, the scanning speed, and the scanning number to be set. Since channels are produced by moving the laser beam along a single direction, the f-theta lens allows the beam to ablate the PMMA surface perpendicularly. This enables the same spot dimensions on the whole working area and a uniform removal rate.

**Figure 2:**
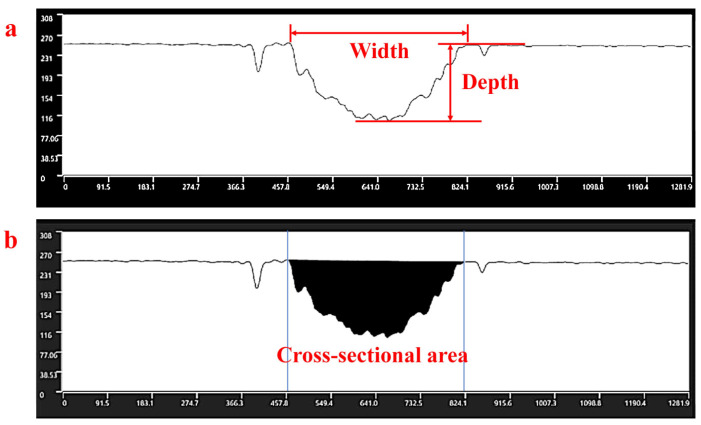
Measurement method of the channel profile using LSCM: (**a**) width and depth; (**b**) cross-sectional area.

The size of the PMMA samples used in the experiments is 60 mm × 60 mm × 2 mm. The cleaned PMMA sample is placed on an adjustable platform to allow the setting of the focal point onto the PMMA surface. The ablation behavior of the laser machining straight channels on PMMA has been studied based on various laser fluences, scanning speeds and scanning numbers. The profiles and morphologies of the channels were obtained using laser scanning confocal microscopy (LSCM) and scanning electron microscopy (SEM). The depth, width, and cross-sectional area of the channel are measured using LSCM, and the measurement method is shown in Figure 2. The measurement tests are conducted 5–7 times at different length positions in each channel. To obtain an accurate cross-sectional profile of the microchannel, the samples containing channels are cut across the channel length using a ps pulsed laser. The cross-sectioned samples are polished using a variety of mesh sizes of emery papers and velvet cloth for a good surface finish. Then, accurate microscopic images of the cross-sectioned profile of the channel are captured using LSCM. A less than 5% error is found between the results obtained from direct measurements using LSCM and the results from cutting and polishing. 

The ablation mass of PMMA is measured using a high-precision electronic balance with an accuracy of 0.1 mg. The measurement steps include the following. First, the sample is subjected to ultrasonic cleaning using purified water. Then, the weight of the sample after drying is measured using the balance. Some parallel microchannels are fabricated on the sample based on the same laser parameters, and the weight of the sample after machining is measured. Then, the ablation mass under the laser parameters is calculated by dividing the weight difference of the sample by the number of the channels on the sample. Finally, the above process is repeated to gain the influence of different laser parameters on the ablation mass. The measurements and calculations of the ablation mass under the same laser parameters are repeated 3 times.

## 3. Experimental Results and Discussion

### 3.1. Laser Ablation Threshold

In the experiments, the laser power is a constant value. Considering that the beam spot size remains unchanged on the surface of the PMMA sample, the laser power density irradiated on the PMMA is a constant value. The total laser energy acted on the PMMA is related to the total laser irradiation time. Here, the laser power density I, the total laser energy density $F_{\mathrm{tot}}$, the total laser energy $E_{\mathrm{tot}}$, and the total laser irradiation time $t_{\mathrm{tot}}$ are represented using the following equations [30,31,40]:

(1) $$I=\frac{P}{\pi {R_{0}}^{2}}$$

(2) $$F_{\mathrm{tot}}=\frac{nP}{2vR_{0}}$$

(3) $$E_{\mathrm{tot}}=Pt_{\mathrm{tot}}$$

(4) $$t_{\mathrm{tot}}=\frac{nL}{v}$$

where the laser power P=7.8 W, the spot radius $R_{0}=70$ µm, and the channel length fabricated by laser ablation L=20 mm. The laser scanning speed v and scanning number n vary in the experiments. For the case of a single-pass laser process, n=1 and $F_{\mathrm{tot}}$ can be represented as $F_{\sin}$.

**Figure 3:**
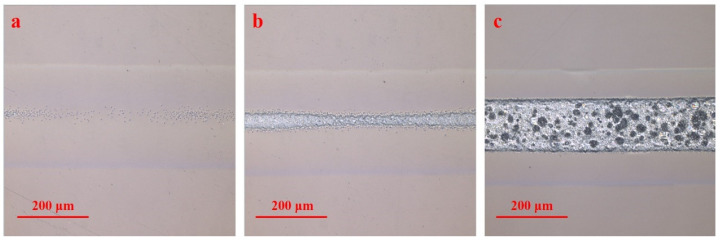
Ablation morphology on the surface of PMMA obtained with a single-pass laser scan with the following scanning speeds and laser energy densities: (**a**) 2700 mm/s, 2.06 J/cm^2^; (**b**) 2600 mm/s, 2.14 J/cm^2^; (**c**) 1800 mm/s, 3.10 J/cm^2^.

Figure 3 shows the microscope images of the laser ablating PMMA surface with a single-pass laser scan at a scanning speed from 1800 mm/s to 2700 mm/s. According to Equation (2), the laser energy density $F_{\sin}$ increases from 2.06 J/cm^2^ to 3.10 J/cm^2^ with the scanning speed decreasing from 2700 mm/s to 1800 mm/s. When the laser energy density is 2.06 J/cm^2^, small pores appear at the laser irradiation center on the surface of PMMA as shown in Figure 3a. The irradiated surface is generally flat with a slight outward expansion. When the laser energy density is 2.14 J/cm^2^, obvious ablation marks appear at the laser irradiation center as shown in Figure 3b. The ablated area is no longer flat, and some small bumps appear. When the laser energy density is 3.10 J/cm^2^, the ablated area is widened, and a large number of pores of various sizes and depths appear in the ablated area as shown in Figure 3c. In the ablated area, the pores with black spots have a greater depth than those with white spots. Meanwhile, partial surface retreat phenomenon occurs in the ablated area, and bulges appear at the edges of the ablated area.

**Figure 4:**
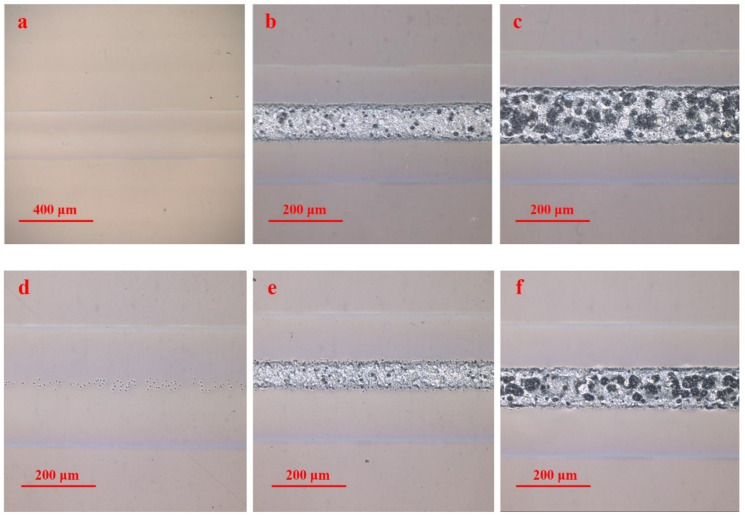
Ablation morphology on the surface of PMMA obtained with a multi-pass laser scanning system with the following scanning speeds, scanning numbers, and total laser energy density: (**a**) 3000 mm/s, 1 pass, 1.86 J/cm^2^; (**b**) 3000 mm/s, 2 pass, 3.71 J/cm^2^; (**c**) 3000 mm/s, 3 pass, 5.57 J/cm^2^; (**d**) 6000 mm/s, 5 pass, 4.64 J/cm^2^; (**e**) 6000 mm/s, 6 pass, 5.57 J/cm^2^; (**f**) 6000 mm/s, 7 pass, 6.50 J/cm^2^.

Figure 4 shows the microscope images of the laser ablating PMMA surface obtained with a multi-pass laser scan at scanning speeds of 3000 mm/s and 6000 mm/s. In the case of 3000 mm/s, the laser energy density $F_{\mathrm{tot}}$ increases from 1.86 J/cm^2^ to 5.57 J/cm^2^ as the scanning number increases from 1 pass to 3 passes. In the case of 6000 mm/s, the laser energy density increases from 4.64 J/cm^2^ to 6.50 J/cm^2^ as the scanning number increases from 5 passes to 7 passes. It can be found that the irradiated surface undergoes a process of no ablation, the occurrence of ablation, and surface retreat as the total laser energy density $F_{\mathrm{tot}}$ increases. The ablation morphology using a multi-pass laser scanning system is similar to that obtained with a single-pass laser scan. However, the ablation threshold and the surface retreat threshold vary with the changes in the scanning speed and the scanning number.

The ablation threshold and the surface retreat threshold with different scan modes are shown in Table 1. Table 1 shows that the single-pass mode has a lower ablation threshold and a lower surface retreat threshold compared to the multi-pass mode. For the multi-pass mode, a faster laser scanning speed, or a lower laser energy density of a single-pass $F_{\sin}$, causes a higher ablation threshold and a higher surface retreat threshold. Meanwhile, by increasing the scanning number, the multi-pass mode with a low $F_{\sin}$ can achieve the same ablation effect as the laser scan mode with a high $F_{\sin}$ [39]. These phenomena are attributed to the incubation effect of laser ablating PMMA.

The authors believe that regardless of whether it is a continuous CO_2_ laser or a ns pulsed CO_2_ laser, the machining channels on PMMA mainly utilize the photothermal effect of the interaction mechanism between the laser and PMMA. The laser energy absorbed by PMMA is converted into material thermal energy. When sufficient thermal energy is deposited onto the material, a series of reactions such as pyrolysis, ablation, and gasification occur, ultimately leading to the material removal from the channel. Meanwhile, there are other mechanisms such as heat conduction, surface convective cooling, and environmental radiation that lead to a decrease in material temperature during the ablation process. Therefore, decreasing the laser scanning speed and increasing the pulse overlap rate can promote the deposition of material thermal energy in a short period of time until complete material ablation is achieved, thereby improving the ablation efficiency of the laser energy. On the other hand, under the same total laser energy density $F_{\mathrm{tot}}$, increasing the laser scanning speed and the scanning number and decreasing the pulse overlap rate may lead to more heat loss and dissipation by thermal conduction, surface convective cooling, and environmental radiation, thereby increasing the ablation threshold and surface retreat threshold.

### 3.2. Thermal Decomposition Behavior of PMMA

**Figure 5:**
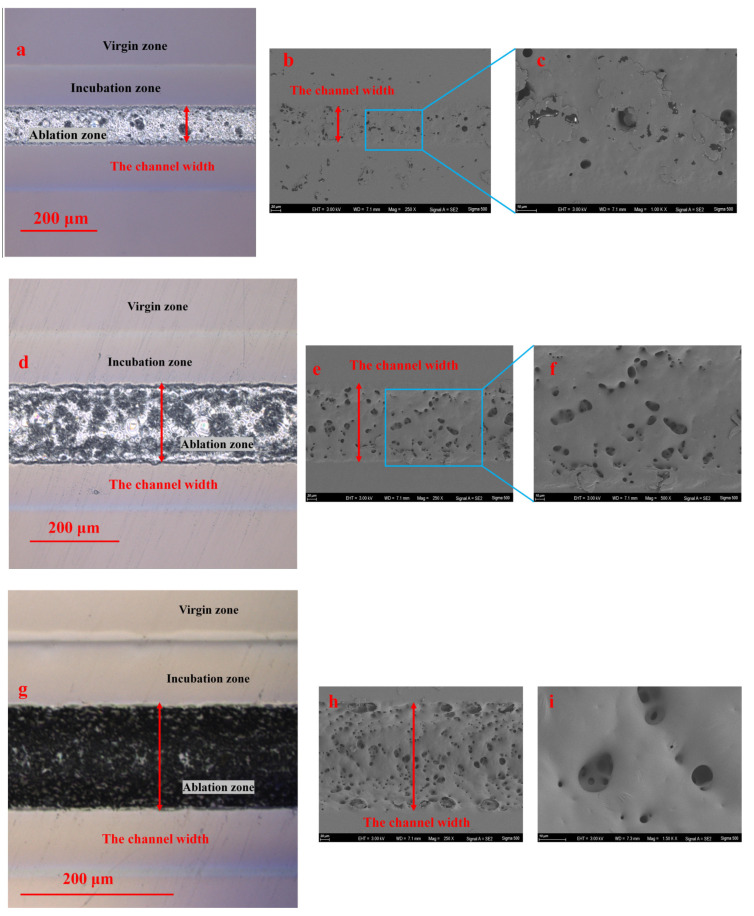
Ablation morphology images obtained using LSCM and SEM: (1) Scanning speed of 3000 mm/s for 2 passes: (**a**) LSCM, 20×; (**b**) SEM, 250X; (**c**) SEM, 1000×; (2) Scanning speed of 3000 mm/s for 4 passes: (**d**) LSCM, 20×; (**e**) SEM, 250×; (**f**) SEM, 500×; (3) Scanning speed of 3000 mm/s for 12 passes: (**g**) LSCM, 20×; (**h**) SEM, 250×; (**i**) SEM, 1500×.

Figure 5a,d,g show that the surface of PMMA after laser ablation can be divided into three regions, which are called the ablation zone, incubation zone, and virgin zone in this paper. In the ablation zone, there are dense pores and microcavity, while the entire surface retreats. The virgin zone is far away from the laser irradiation area. There is no visible change between the virgin zone and the unirradiated sample. The incubation zone is located between the ablation zone and the virgin zone, and there are clear boundaries between them. In the incubation zone, the material surface slightly expands outward. Meanwhile, some small pores appear at the boundary between the ablation zone and the incubation zone. It is indicated that the polymer in the virgin zone does not undergo the thermal decomposition reaction. The polymer in the incubation zone undergoes the early stages of the thermal decomposition reaction. Moreover, a part of polymer in the ablation zone undergoes the complete thermal decomposition reaction and is ultimately removed.

The bulge formation noted at the boundary between the ablation zone and the incubation zone is probably due to two possible reasons [34]. One possible reason is the softening and expansion of the polymer after laser irradiation. A second possible reason is that the molten polymer is ejected from the center to the edges of the ablation zone by gaseous products from the decomposition reaction.

PMMA melts at 130 °C, and it almost does not undergo decomposition reactions up to 200 °C. When the temperature is between 220 °C and 300 °C, PMMA mainly undergoes a depolymerization reaction, which decomposes PMMA into methyl methacrylate (MMA) monomers. Within this temperature range, the depolymerization reaction accounts for 95% of the mass decrease in PMMA. Meanwhile, due to the boiling point of MMA being 100 °C, the MMA generated from the depolymerization reaction is maintained as a volatile gas during the entire decomposition reaction. When the temperature is between 360 °C and 400 °C, the MMA monomer further decomposes to yield small molecular gaseous products, such as methane (CH_4_), methanol (CH_4_O), formaldehyde (CH_2_O), etc. Finally, the gaseous products undergo combustion to yield the final products, such as CO_2_, CO, H_2_O, and energy [41].

It can be found that the density and size of pores inside the channel increase as the number of laser scans increases from 2 to 12 as shown in Figure 5b,c,e,f,h,i. In Figure 5c, a sheet-like PMMA is noted that has melted and resolidified inside and outside the channel at a scanning speed of 3000 mm/s with 2 passes of the laser scan. It is indicated that the low temperature of PMMA leads to a low depolymerization reaction level due to a low total laser energy density $F_{\mathrm{tot}}$. Hence, a portion of the polymer in the ablation zone melts and resolidifies without undergoing the depolymerization reaction. A part of the molten polymer leaves the channel due to splashing and then resolidifies in the incubation zone. In Figure 5f,i, small pores appear inside the large pores. The extent of depolymerization reaction is high with a high number of laser scans. Only a small amount of resolidified sheet-like PMMA appears in the channel, with most concentrating at the boundary between the ablation zone and the incubation zone as shown in Figure 5e,h. It is indicated that the PMMA in the ablation zone center undergoes a thorough depolymerization reaction as the total laser energy density $F_{\mathrm{tot}}$ increases. A large amount of polymer directly transitions from solid to gas due to incubation effect from the previous laser scans. Therefore, it is found that the channel depth and the material porosity of the ablation zone increase as the total laser energy density $F_{\mathrm{tot}}$ increases. Meanwhile, it reveals that an increase in the scanning number improves the decomposition reactions of the PMMA under the same laser energy density of a single-pass $F_{\sin}$.

### 3.3. The Profile, Width, and Depth of the Channel

**Figure 6:**
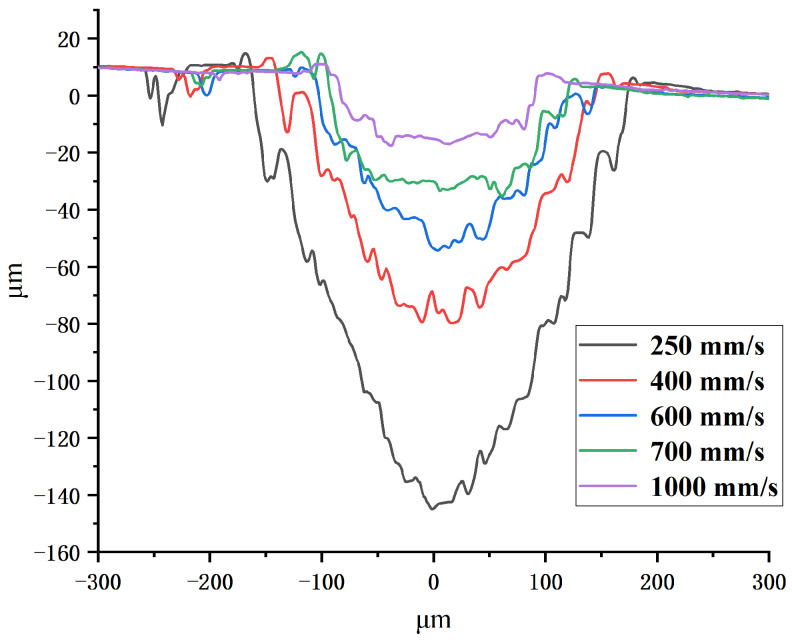
The cross-sectional profile of the channel obtained in single-pass mode at a scanning speed of 250 mm/s to 1000 mm/s.

**Figure 7:**
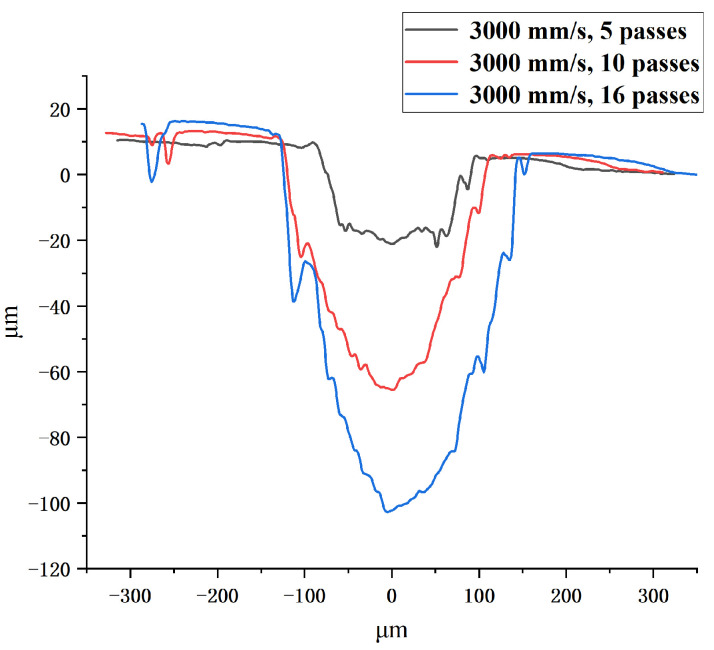
The cross-sectional profile of the channel obtained in multi-pass mode at a scanning speed of 3000 mm/s from 5 passes to 16 passes.

Figure 6 shows that the cross-sectional profile of the channel is approximately V-shaped at a scanning speed of 250 mm/s to 600 mm/s in single-pass mode. The cross-sectional profile of the channel becomes shallow and gentle as the scanning speed increases. Then, the cross-sectional profile of the channel become approximately trapezoidal at a scanning speed of 700 mm/s to 1000 mm/s. It can be found that the depth, width, and aspect ratio of the channel increase as the laser scanning speed decreases. Figure 7 shows that the cross-sectional profile of the channel gradually changes from trapezoidal to V-shaped as the scanning number increases from 5 to 16 at a scanning speed of 3000 mm/s. It can be found that the depth, width, and aspect ratio of the channel increase as the number of laser scans increases. The result indicates that the higher the total laser energy density $F_{\mathrm{tot}}$ is, the greater the aspect ratio of the channel, and the cross-section profile of the channel is closer to V-shaped. The smaller the total laser energy density $F_{\mathrm{tot}}$ is, the smaller the aspect ratio of the channel, and the cross-section profile of the channel is closer to trapezoidal.

**Figure 8:**
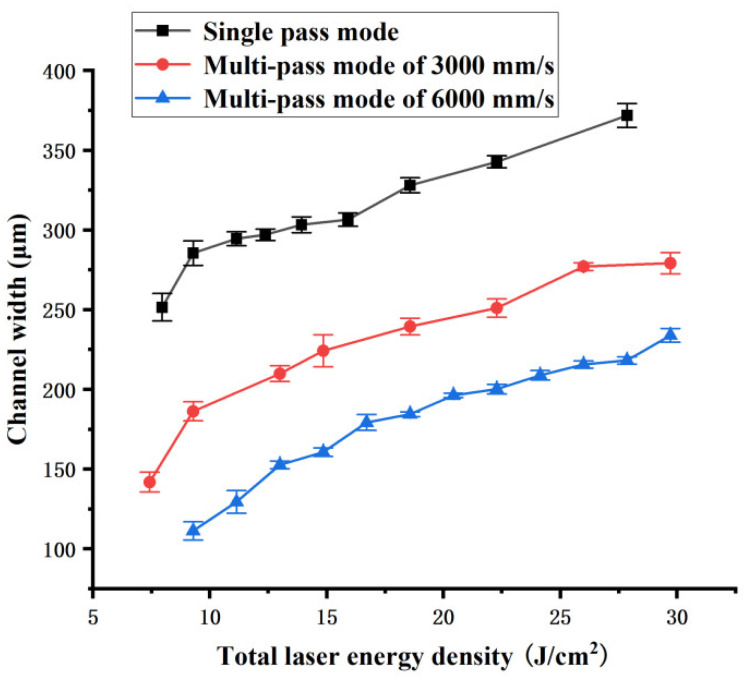
Channel width dependence on the total laser energy density of different laser scan modes.

**Figure 9:**
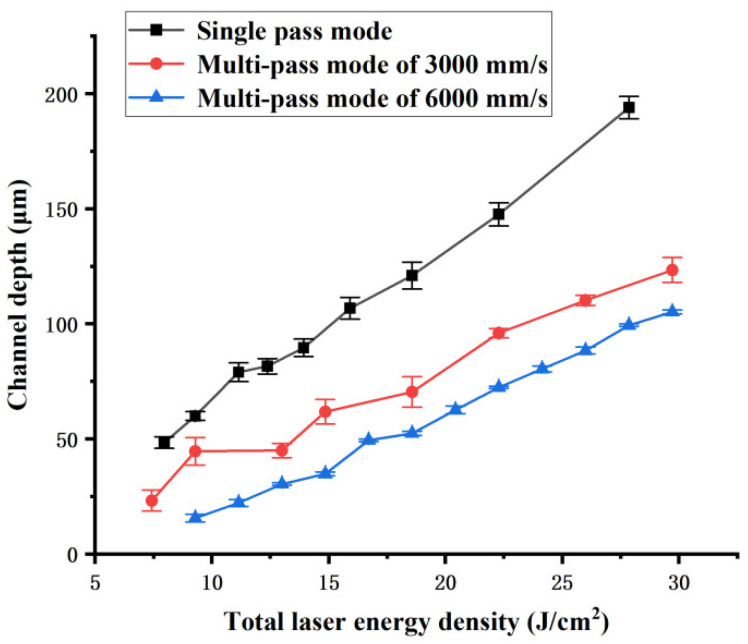
Channel depth dependence on the total laser energy density of different laser scan modes.

The influence of the total laser energy density $F_{\mathrm{tot}}$ on channel width and depth are shown in Figure 8 and Figure 9, respectively. They show that the width and depth of the channel ablated by different single-pass and multi-pass laser scan modes increase as the total laser energy density $F_{\mathrm{tot}}$ increases. The width and the depth of the channel obtained in single-pass mode are much larger than those obtained in multi-pass mode under the same total laser energy density $F_{\mathrm{tot}}$. Meanwhile, the width and depth of channel obtained in a multi-pass mode of 3000 mm/s are larger than those obtained at 6000 mm/s under the same total laser energy density $F_{\mathrm{tot}}$. As mentioned earlier, according to the theory of laser ablation and thermal deposition, under the same total laser energy density $F_{\mathrm{tot}}$, the higher the laser energy density of single pass $F_{\sin}$ is, the higher the utilization efficiency of laser energy for material ablation, and the less heat dissipated during the channel manufacturing process. Therefore, the channel width and depth of single-pass mode are greater than those of multi-pass mode. The channel width and depth obtained at 3000 mm/s are greater than those obtained at 6000 mm/s under the same total laser energy density.

In addition, it can be found that the channel width under many laser scan modes is much larger than the laser spot diameter. The authors believe that in the final stage of the PMMA thermal decomposition reaction, the combustion exothermic reaction of small molecule gas products provides additional energy. This allows the surface material outside the laser spot to melt and thermally decompose, thereby expanding the channel width outward.

### 3.4. Volume Ablation Rate and Mass Ablation Rate

The volume of the channel V can be expressed as follows:

(5) V=SL

where S is cross-sectional area of the channel. The volume ablation rate can be expressed as follows:

(6) $$\eta_{V}=\frac{V}{E_{\mathrm{tot}}}$$

Substituting Equations (3)–(5) into Equation (6), the volume ablation rate $\eta_{V}$ can be written as follows:

(7) $$\eta_{V}=\frac{Sv}{Pn}$$

The mass ablation rate $\eta_{m}$ of the channel can be calculated as follows:

(8) $$\eta_{m}=\frac{\Delta m}{E_{\mathrm{tot}}}$$

where Δm is the mass loss caused by laser ablation.

Substituting Equations (3) and (4) into Equation (8), the mass ablation rate $\eta_{m}$ can be written as follows:

(9) $$\eta_{m}=\frac{\Delta mv}{PnL}$$

**Figure 10:**
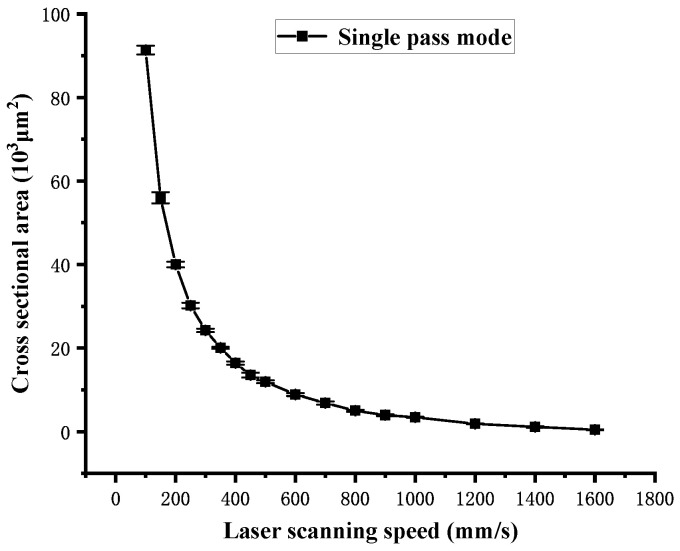
Cross-sectional area dependence on the laser scanning speed in single-pass mode.

**Figure 11:**
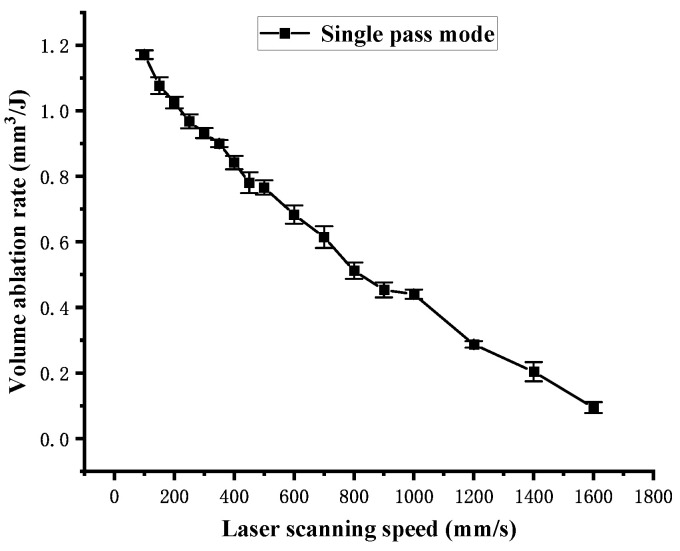
Volume ablation rate dependence on the laser scanning speed in single-pass mode.

Figure 10 shows the influence of the laser scanning speed on the cross-sectional area S of the channel obtained in single-pass mode. As the laser scanning speed increases, the cross-sectional area S decreases. According to Equation (2), as the laser scanning speed increases from 100 mm/s to 300 mm/s, the laser energy density $F_{\sin}$ acting on the material surface decreases by 37.14 J/cm^2^. However, as the laser scanning speed increases from 300 mm/s to 1600 mm/s, the laser energy density $F_{\sin}$ only decreases by 15.09 J/cm^2^. Therefore, the curve in Figure 10 shows a sharp change between 100 mm/s and 300 mm/s, following a slow change between 300 mm/s and 1600 mm/s. The volume ablation rate is calculated using Equation (7), and the influence of the laser scanning speed on the volume ablation rate in single-pass mode is shown in Figure 11. The curve in Figure 11 is approximately linear. In Figure 10, it can be found that there is an approximate functional relationship between the scanning speed and the cross-sectional area, which is noted as follows:

(10) $$S=\frac{\chi }{v^{2}}$$

where χ is a constant. Substituting Equation (10) into Equation (7), the volume ablation rate of single pass mode can be written as follows:

(11) $$\eta_{V}=\frac{\chi }{Pv}$$

Due to the laser power P in the experiment being a constant, the volume ablation rate is inversely proportional to the scanning speed as shown in Figure 11. It indicates that the volume ablation rate increases as the laser scanning speed decreases. The utilization efficiency of the laser energy used for material ablation is also inversely proportional to the scanning speed.

According to incubation effect, the higher the pulse overlap rate and the lower the laser scanning speed are, the lower the laser ablation threshold of polymer material, which makes it more susceptible to ablation. The incubation effect is consistent given the theory of laser energy thermal deposition. It reveals that the higher the heat flux density from a higher $F_{\sin}$ is, the faster the thermal energy deposition inside the material, and the faster the material is ablated. It leads to a lower laser ablation threshold due to rapid ablation reducing thermal dissipation by reducing the interaction time between the laser and the irradiated material.

**Figure 12:**
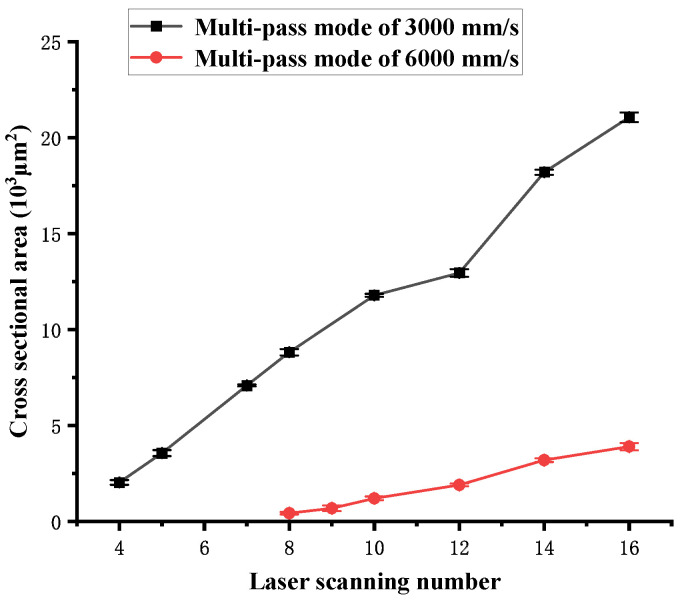
Cross-sectional area dependence on the number of laser scans in multi-pass mode.

Figure 12 shows the influence of the number of laser scans on the cross-sectional area S of the channel obtained in multi-pass mode. As the number of laser scans increases, the cross-sectional area increases. No strict linear relationship is noted between the scanning number and the cross-sectional area of the channel. The cross-sectional area of 3000 mm/s is larger than that of 6000 mm/s under the same laser scan passes. Although the laser energy density $F_{\mathrm{tot}}$ of 3000 mm/s is twice that obtained at 6000 mm/s under the same scanning number, the cross-sectional area of 3000 mm/s is more than 5.4 times that obtained at 6000 mm/s. It is attributed to the incubation effect. A higher $F_{\sin}$ causes a lower ablation threshold, leading to a faster channel volume increase.

**Figure 13:**
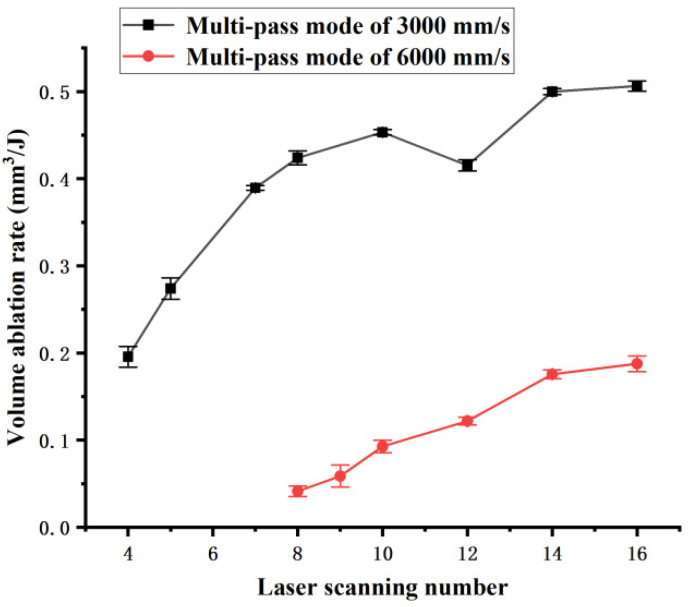
Volume ablation rate dependence on the number of laser scans in multi-pass mode.

Figure 13 shows the influence of the number of laser scans on the volume ablation rate of the channel for the multi-pass mode. It is found that the overall volume ablation rate increases as the number of laser scans increases, but the curve obtained at 3000 mm/s also shows some downward trends at some specific points. The volume ablation rate obtained at 3000 mm/s is more than 2.6 times that obtained at 6000 mm/s. The curve obtained at 3000 mm/s rapidly increases in the initial stage, indicating a rapid increase in the volume ablation rate due to the incubation effect caused by the early laser scan passes. Compared with the virgin material, the polymer, which has undergone an early depolymerization reaction after some passes of laser scanning, is more susceptible to ablation. Therefore, the early laser scan passes produce a small ablation zone and a large incubation zone, showing a low volume ablation rate. As the number of laser scans increases, the polymer in the incubation zone is ablated rapidly, leading to a rapid increase in the volume ablation rate.

**Figure 14:**
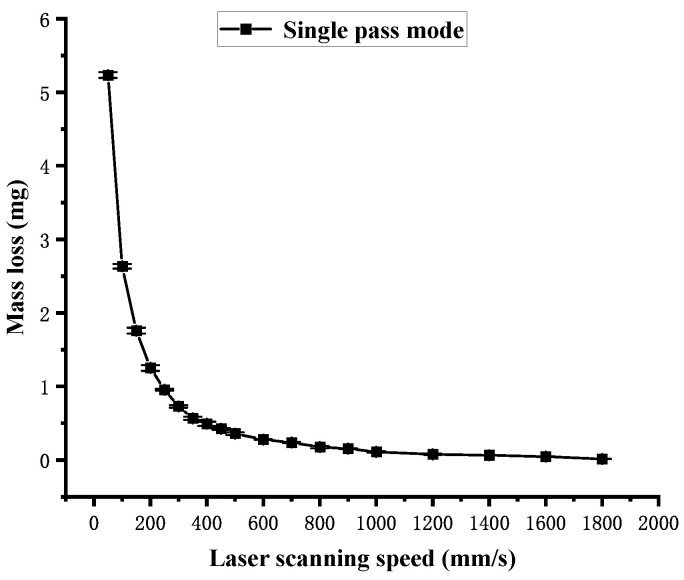
Mass loss dependence on the laser scanning speed in single-pass mode.

**Figure 15:**
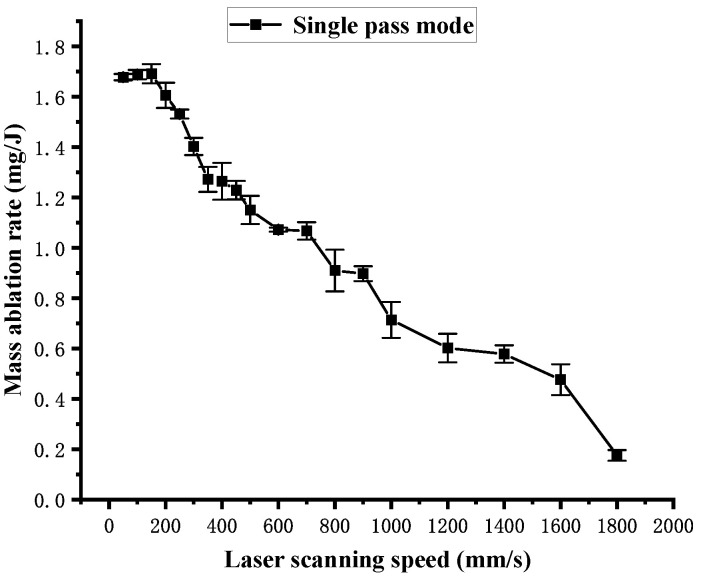
Mass ablation rate dependence on the laser scanning speed in single-pass mode.

Figure 14 shows the influence of the laser scanning speed on the mass loss obtained in single-pass mode. It can be observed that the mass loss decreases as the laser scanning speed increases. Similar to the curve in Figure 10, the curve in Figure 14 shows a sharp change between 50 mm/s and 300 mm/s, following a slow change between 300 mm/s and 1800 mm/s. It is also attributed to the change in the laser energy density $F_{\sin}$ in different speed ranges. The mass ablation rate is calculated using Equation (9), and the influence of the laser scanning speed on the mass ablation rate is shown in Figure 15. It shows that the overall mass ablation rate decreases as the laser scanning speed increases, and this curve is similar to the curve in Figure 13. However, the curve in Figure 15 has an upward trend or a plateau at some specific points.

**Figure 16:**
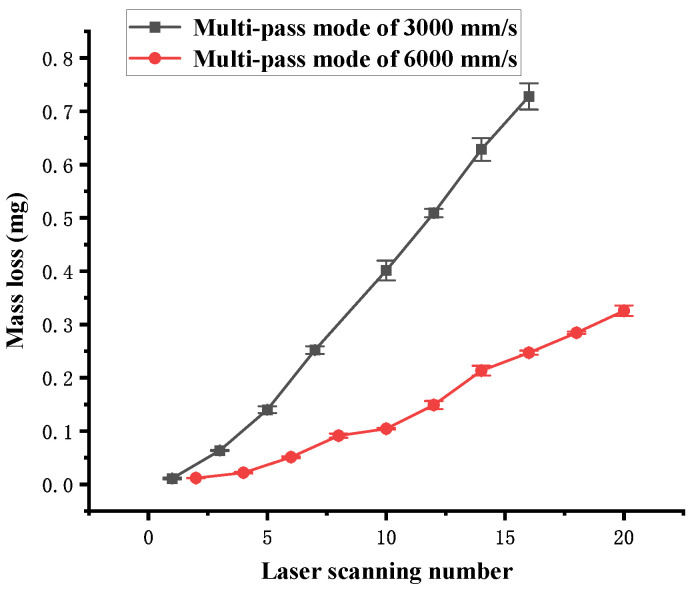
Mass loss dependence on the number of scans in multi-pass mode.

Figure 16 shows the influence of the number of laser scans on the mass loss in multi-pass mode. As the number of laser scans increases, the mass loss increases. A linear relationship is noted between the scanning number and the mass loss of the channel. Although the laser energy density $F_{\mathrm{tot}}$ of 3000 mm/s is twice that of 6000 mm/s under the same number of scans, the mass ablation rate of 3000 mm/s far exceeds twice that of 6000 mm/s, even reaching 3.8 times when the number of scans is greater than 5. It is also attributed to the incubation effect, which is similar to the relationship observed in Figure 12.

**Figure 17:**
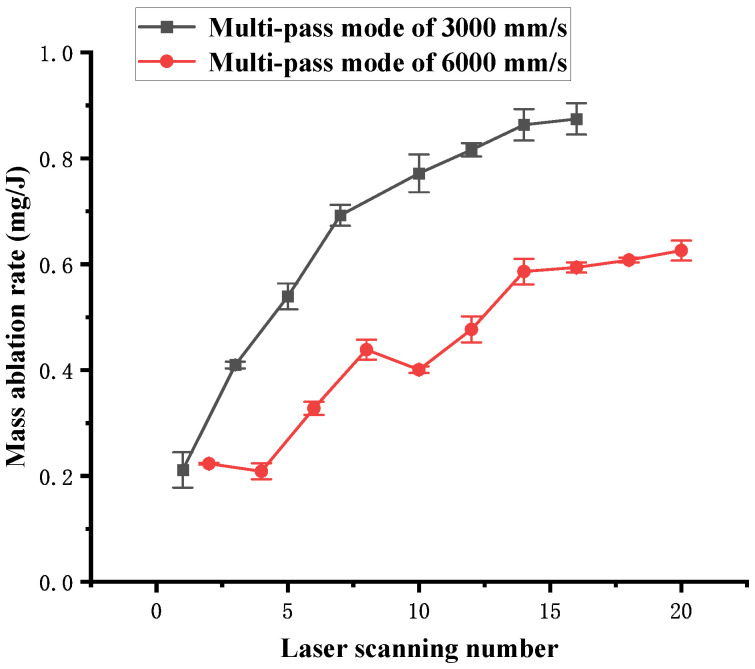
Mass ablation rate dependence on the number of scans in multi-pass mode.

Figure 17 shows the influence of the laser scanning number on the mass ablation rate in the multi-pass mode. The curve obtained at 3000 mm/s rises rapidly at first and then rises slowly as the scanning number increases. However, the curve obtained at 6000 mm/s generally rises as the scanning number increases and exhibits a downward trend at some special points.

Figure 10, Figure 11, Figure 12, Figure 13, Figure 14, Figure 15, Figure 16 and Figure 17 indicate that the influence of the scanning speed on the volume ablation rate and the mass ablation rate is greater than that noted for the number of scans. Based on the calculations, the volume ablation rate and the mass ablation rate obtained in single-pass mode are larger than those obtained in multi-pass mode under the same total laser energy density. Due to the fact that the thermal decomposition reaction of PMMA is divided into several stages, there is no linear relationship between the reduction of material volume and mass and the absorbed laser energy. In some cases, the polymer has absorbed an amount of laser energy and accumulated a large of thermal energy; however, the changes in volume and mass may not be significant because the material has not undergone a sufficient depolymerization reaction. It leads to a plateau, a slower change or a reverse in the curves of the volume ablation rate and mass ablation rate in Figure 11, Figure 13, Figure 15 and Figure 17. When the laser scanning number increases and the scanning speed decreases, the heat input of laser irradiation increases, allowing the partially molten and incompletely depolymerized polymer to obtain sufficient energy for further decomposition and gasification. It causes a rapid decrease in the volume and mass of the irradiated material, leading to a rapid change in the volume ablation rate and mass ablation rate curves.

## 4. Simulation Calculation

**Figure 18:**
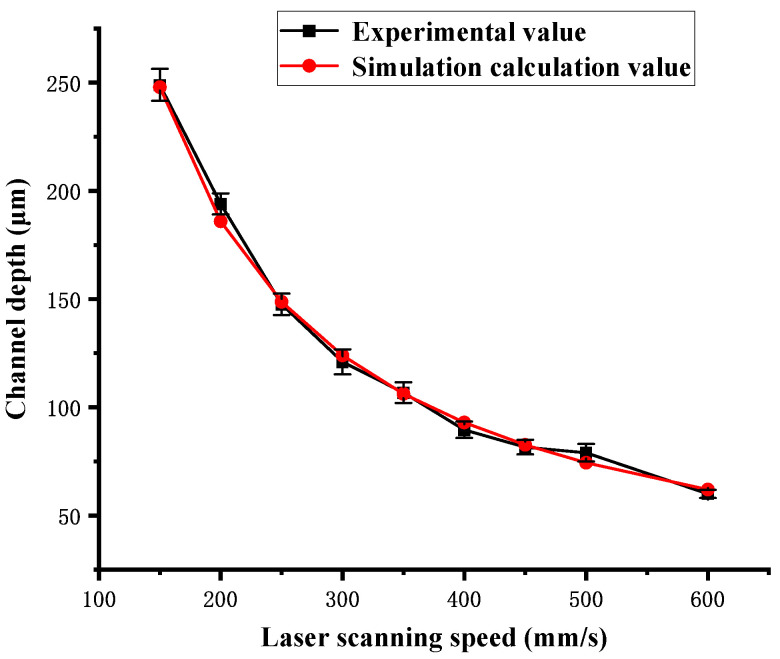
Dependence of channel depth of the experimental values and the simulation calculation values on laser scanning speed in single-pass mode.

The effectiveness of the simulation models proposed by Yuan [30] and Prakash [31] et al. has been validated in the low-power laser manufacturing of PMMA microchannels. This paper combines existing simulation models to derive the profile expression of the channel cross-section after a single-pass laser scan. The laser power density at different positions on the laser focal plane can be expressed as follows:

(12) $$I_{s}=\frac{\beta P}{\pi {R_{0}}^{2}}e^{-\frac{\beta (x^{2}+y^{2})}{{R_{0}}^{2}}}$$

where β is the laser Gaussian shape coefficient. Here, x and y represent the position coordinates along the channel length and the channel width directions, respectively. The channel depth $z_{\max}$ and the depth z at different positions of the channel cross-section can be expressed respectively as follows:

(13) $$z_{\max}=\frac{\alpha }{\rho (C_{p}\Delta T+H_{L})}\sqrt{\frac{\beta }{\pi {R_{0}}^{2}}}\frac{P}{v}$$

(14) $$z=z_{\max}e^{-\frac{\beta y^{2}}{{R_{0}}^{2}}}$$

where the laser absorption coefficient α is 0.95, the PMMA density ρ is 1070 kg/m^3^, the specific heat capacity $C_{p}$ is 1466 J/(kg K), the temperature difference ΔT from room temperature (20 °C) to completion of the depolymerization reaction (393 °C) is 373 K, and the gasification latent heat $H_{L}$ is 1800 kJ/kg [31,42]. According to Equation (13), a great consistency in channel depth is achieved between the experimental and simulation results as the laser scanning speed varies from 150 mm/s to 600 mm/s when β is equal to 2.444. The experimental and simulation results of the channel depth are shown in Figure 18. The error percentages between the experimental and simulation results are shown in Table 2. The error percentage of the channel depth varies from 0.44% to 5.9%.

However, this calculation model ignores the effects of heat conduction, surface convective cooling, environmental radiation, and heat dissipation, so it is not suitable for single-pass laser ablation with a low energy density $F_{\sin}$, and multi-pass laser ablation is severely affected by heat dissipation. Meanwhile, the experimental channel depth is greater than the calculated channel depth when the laser energy density of single-pass $F_{\sin}$ is too high, such as 55.7 J/cm^2^ at the scanning speed of 100 mm/s. It is hypothesized that the gasification latent heat used in the calculation model does not include the contribution of exothermic oxidation of the small molecule organic gas in the last stage of the decomposition reaction.

**Figure 19:**
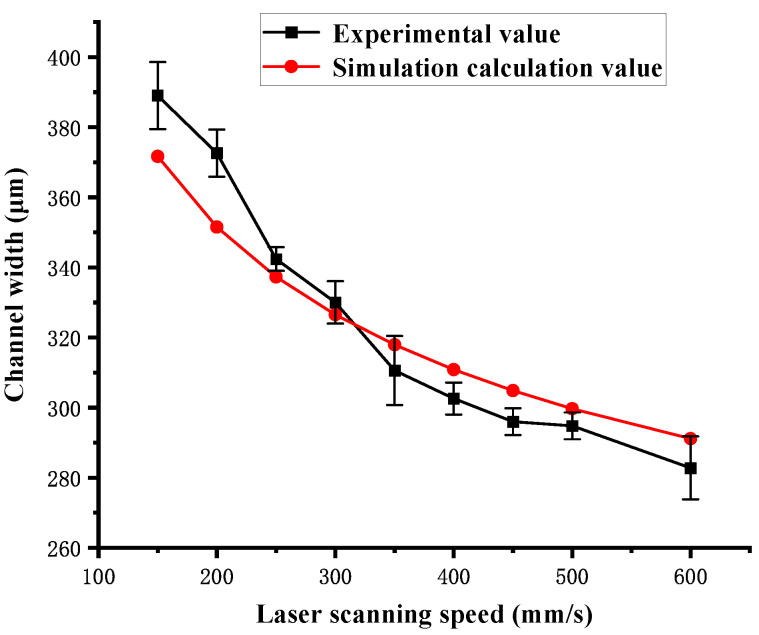
Dependence of channel width of the experimental values and the simulation calculation values on laser scanning speed in single-pass mode.

When the laser scanning speed varies from 150 mm/s to 600 mm/s, the channel width is larger than the laser spot diameter. An approximate reciprocal relationship exists between the channel width and the logarithm of the scanning speed. The relationship can be written as follows:

(15) $$w[µm]=\frac{c}{\ln(v[s/\mathrm{mm}])}$$

where w is the channel width, and c is the correlation coefficient. According to Equation (15), a great consistency in channel width is achieved between the experimental and simulation results when c is equal to 1862.37. The experimental and simulation results of the channel width are shown in Figure 19. The error percentages between the experimental and simulation results are shown in Table 2. The error percentage of the channel width varies from 1.1% to 5.7%. When a high-power laser is used to fabricate channels on the surface of PMMA, the mechanism of channel width variation involves complex heat transfer and thermal decomposition reaction processes. Thus, the channel width cannot be calculated using the simplified models of Yuan [30] and Prakash et al. [31].

Combining Equations (13)–(15), a corrected expression describing the cross-sectional profile of the channel obtained in single-pass mode can be obtain as follows:

(16) $$z=\frac{\alpha }{\rho (C_{p}\Delta T+H_{L})}\sqrt{\frac{\beta }{\pi {R_{0}}^{2}}}\frac{P}{v}e^{-\frac{\beta y^{2}}{w^{2}}}$$

**Figure 20:**
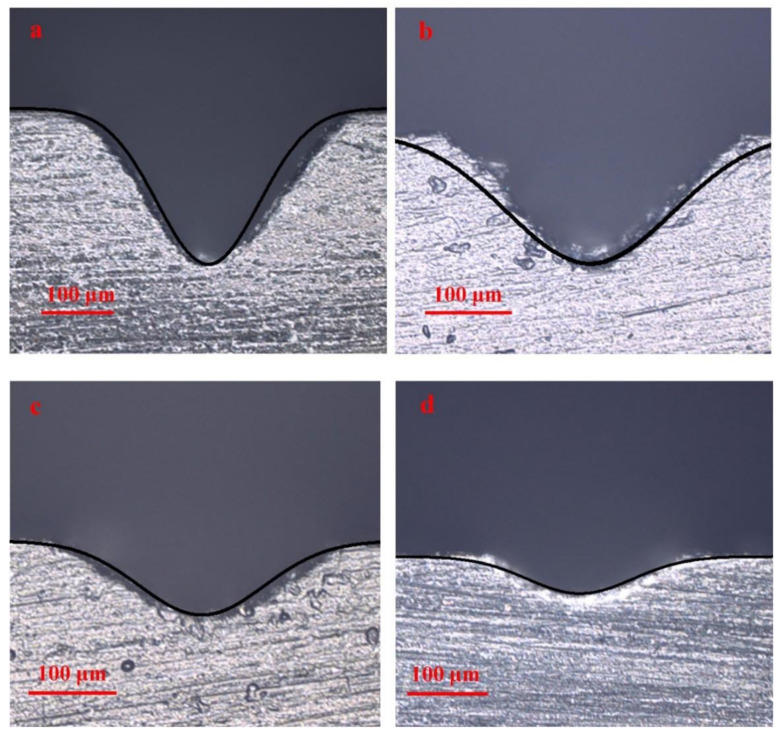
The cross-sectional profile of the channels obtained by using different laser scanning speeds: (**a**) 150 mm/s; (**b**) 250 mm/s; (**c**) 350 mm/s; (**d**) 600 mm/s.

The cross-sectional profile of the channel is calculated using Equation (16), and the experimental and simulation results are shown in Figure 20. A great consistency in channel profile is achieved between the experimental and simulation results. Meanwhile, the effectiveness of the model proposed in this paper has been validated when the laser scanning speed varies from 150 mm/s to 600 mm/s.

## 5. Conclusions

The ablation threshold and the ablation morphology of PMMA are investigated under single-pass laser scanning and multi-pass laser scanning, separately. It is found that a higher laser energy density of a single-pass laser scan causes a lower ablation threshold. The ablated surface can be divided into three regions: the ablation zone, incubation zone, and virgin zone. The ablation process of PMMA is mainly attributed to the thermal decomposition reactions, as well as the splashing of molten polymer.

The influences of laser scanning speed, scanning number, and energy density on the channel profile are investigated. The depth, width, aspect ratio, volume ablation rate, and mass ablation rate of the channel increase as the laser scanning speed decreases and the laser scanning number increases. Under the same total laser energy density, a higher laser energy density of a single-pass scan causes a larger channel width, depth, volume ablation rate, and mass ablation rate.

The change in the ablation threshold and the differences in the ablation results under the same total laser energy density are attributed to the incubation effect, which is caused by the thermal deposition of laser energy in the polymer. When the laser energy density of a single-pass scan is high enough, less heat dissipation causes more material ablation, leading to a lower ablation threshold and a higher ablation efficiency of the microchannel with laser machining.

Finally, an optimized simulation model is proposed that is used to solve the problem of obtained a channel width greater than spot diameter with single-pass laser scanning. A great consistency in the depth, width, and cross-sectional profile of the channel is achieved between the experimental and simulation results. The error percentage between the experimental and simulation results varies from 0.44% to 5.9%.

## Figures and Tables

**Table 1:** The ablation threshold and the surface retreat threshold of different laser scan modes.

Laser Scan Mode	Ablation Threshold (J/cm^2^)	Surface Retreat Threshold (J/cm^2^)
Single pass	2.14	3.10
Multi-pass at 3000 mm/s	3.71	5.57
Multi-pass at 6000 mm/s	5.57	6.50

**Table 2:** The error percentages of the channel depth and width between the experimental and simulation results.

Laser Scanning Speed (mm/s)	Error Percentage of Depth (%)	Error Percentage of Width (%)
150	0.44	4.5
200	4.2	5.7
250	0.77	1.5
300	2.4	1.1
350	0.52	2.4
400	3.8	2.7
450	1.3	3.0
500	5.9	1.7
600	3.3	2.9

## Data Availability

The original contributions presented in the study are included in the article, and further inquiries can be directed to the corresponding authors.
